# Recycling of Polypropylene/Polyethylene Blends: Effect of Chain Structure on the Crystallization Behaviors

**DOI:** 10.3390/polym11091456

**Published:** 2019-09-06

**Authors:** Chuanchom Aumnate, Natalie Rudolph, Majid Sarmadi

**Affiliations:** 1 Metallurgy and Material Sciences Research Institute, Chulalongkorn University, Bangkok 10330, Thailand; 2 AREVO, Inc., Milpitas, CA 95035, USA; 3 Textile Science, Department of Design Studies and Materials Science Graduate Program, University of Wisconsin-Madison, Madison, WI 53706, USA

**Keywords:** polymer blends and alloys, recycling, crystallization, crystal structure, kinetics

## Abstract

The combination of high-density polyethylene (HDPE), low-density polyethylene (LDPE) and polypropylene (PP) is frequently found in polymer waste streams. Because of their similar density, they cannot be easily separated from each other in the recycling stream. Blending of PP/ polyethylenes (PEs) in different ratios possibly eliminate the sorting process used in the regular recycling process. PP has fascinating properties such as excellent processability and chemical resistance. However, insufficient flexibility limits its use for specific applications. Blending of PP with relative flexible PEs might improve its flexibility. This is a unique approach for recycling or upcycling, which aims to maintain or improve the properties of recycled materials. The effects of the branched-chain structures of PEs on the crystallization behavior and the related mechanical properties of such blends were investigated. The overall kinetics of crystallization of PP was significantly influenced by the presence of PEs with different branched-chain structures. The presence of LDPE was found to decrease the overall crystallization rate while the addition of HDPE accelerated the crystallization process of the blends. No negative effect on the mechanical performance and the related crystallinity was observed within the studied parameter range.

## 1. Introduction

Plastics are used daily in some applications. A considerable amount of plastic is used in disposable products. The amount of plastic consumed has been growing steadily due to desirable properties such as low density, high strength, ease of manufacturing and low cost. As a result, both industry and private households generate more and more plastic waste. The most prominent concerns are single-use plastic items such as packaging, bags and containers. Unfortunately, appropriate waste management strategies are not developing at the same rate as the increasing levels of plastic wastes. A significant amount of waste does not reach proper disposal sites, instead of littering the landscape, and blowing or washing into the sea, which leads to severe environmental problems. Polyolefins, which have excellent recycling properties, make up over 50% of the non-recycled plastic (high-density polyethylene, HDPE, low-density polyethylene, LDPE and polypropylene, PP). This has motivated the interest in plastic reuse and recycling [1,2,3].

Polyethylenes (PEs) are the most common plastic. Their primary uses are in packaging including plastic bags, plastic films and bottles. PEs are classified according to their polymerization method, density and branching. There are several types of PEs, but the most common use is the high-density polyethylene (HDPE) and the low-density polyethylene (LDPE). HDPE has between five and ten short branches every 1000 carbon atoms. LDPE has the same number of branches as HDPE; however, they are much longer and are usually branched themselves [4,5,6]. Polypropylene (PP) is a linear hydrocarbon polymer with an intermediate level of crystallinity between LDPE and HDPE. Similar to PE in many properties, the presence of the methyl group attached to every other backbone carbon atom can alter the properties in some ways. The tertiary carbon atom can provide a site for oxidation. As a result, PP is less stable than PE. The orientation of each methyl group relative to the adjacent methyl groups has a strong effect on the polymer’s ability to form crystals [7]. Due to its desirable physical properties such as high tensile strength, high stiffness and high chemical resistance, polypropylene (PP) has been widely used as packaging material, for instance as margarine and yogurt containers, bottle caps and microwavable food. However, it shows weak impact strength at low temperatures and is susceptible to environmental stress cracking [8,9]. LDPE waste mostly results from bags and packaging films. Owing to its low mechanical properties and easy processability it is recycled as garbage bags. One possibility to develop alternative applications is blending it with other materials to improve the low mechanical performance of recycled LDPE.

The combination of PP and PEs is frequently found in polymer waste streams. Because of their similar density, PP and PEs cannot be easily separated from each other in the recycling stream [10]. Blends of PP and PEs have become a subject of great economic and research interest, not only to improve the processing and mechanical properties of PP but also to some extent to expand opportunities to recycle these mixed plastics [11,12,13,14,15] Since the 1980s, a considerable amount of work has been focused on the study of mechanical properties of mixed PP and PEs. The effectiveness of blending mainly depends on the miscibility or immiscibility of the blended components. PP and HDPE as well as PP and LDPE are generally considered immiscible in any blending ratio and show a remarkable phase separation during cooling and crystallization, which reflects the end-properties of such blends. In polymer blends, the crystallization process may have a significant influence on its morphological features, thermal and mechanical properties.

Semi-crystalline polymers, PP and PEs, consist of crystallites of different lamellar thickness and degree of perfection. Crystallization kinetics in such polymer blends are very complex in relation to the different crystallization behavior of the two components. These include the production of primary nuclei, formation and spreading of surface nuclei, and inter-diffusion of crystallizable and non-crystallizable chains at the advancing front of the growing crystal [16,17,18]. In general, depending on the nucleation, there are two types of crystallization: heterogeneous and homogeneous crystallization. The crystal fraction in a polymer is a function of both the nucleation rate and the growth rate of the spherulite in the sample. The factors influencing the changes in primary nucleation in polymer blends can be divided into two groups. (i) The properties of the minor phase polymer in the blend, including miscibility, glass transition temperature, ability to crystallize, the temperature range in which crystallization is possible and surface tension of the polymer melt and (ii) the blending/mixing processes. For such blends in which dispersed polymer can crystallize, the size of its inclusions, controlled by the parameters of the mixing process, is one of the most critical factors determining the crystallization kinetics of the blend. For example, the non-equilibrium molecular structure of the PP/LDPE blend can be observed as a result of the strange process of PP crystallization [12]. Accordingly, the crystallization in blends of PP with LDPE and HDPE has been studied. PP has a high degree of crystallinity due to its regular chain structure. Galeski et al. [19] examined the morphology of an isotactic PP (iPP)/LDPE blend and revealed that the LDPE occlusions introduced substantial changes in the internal structure of the iPP spherulites. From a polarizing micrograph, it was seen that the LDPE droplets hindered the spherulite-growing front, causing distinct concavities. The influence of crystallization of a dispersed polymer in the matrix crystallization can probably explain the decrease of spherulite size in nonisothermically crystallized samples of the blends reported for iPP with LDPE [20], and iPP with HDPE [21]. During fast nonisothermal crystallization, a simultaneous crystallization of both components is also possible. Already crystallized inclusions of a dispersed polymer accelerate the crystallization of the matrix acting as nucleating agent and induce the formation of additional spherulites. Finally, the average spherulite radius in those blends becomes smaller than in plain iPP crystallized under the same condition [22]. Razavi-Nouri et al. [23] studied the morphology of PP/linear low-density PE (LLDPE) blends and found that the nucleation densities of the PP spherulites decreased in the presence of the LLDPE. Moreover, LLDPE remained as discrete droplets dispersed throughout the PP spherulites, which indicated the immiscibility of these two components. In the case of PE/PE blends, molten PEs of different type chain structures usually are immiscible. The spherulites of PE with higher *T*_m_, are encapsulated by those of the other PE during crystallization. Thus, once cooled below the crystallization point of one component, the blends’ morphology is fixed within the crystalline structure.

The physical properties of PP and PEs are too similar to enable the detection of such phase separation, which governs the mechanical properties of the end product directly. Likewise, the phase behaviors of PP/PEs blends, are not entirely understood yet. However, the crystallization behavior plays an essential role in determining the crystal structure as well as the phase morphology, which further affects the end-use properties, especially mechanical performance. In this study, the influences of different chain structures PEs on the crystallization behaviors of PP/PEs blends as well as effects on the tensile properties are investigated. The crystallization behaviors, including the kinetics of crystallization as well as the crystal morphology, were studied using the differential scanning calorimetry (DSC) and the polarized optical microscopy techniques, respectively. The gel permeation chromatography (GPC) technique was used to measure the molecular weight and molecular weight distribution of the materials. Also, the blend morphology was analyzed via the scanning electron microscopy (SEM). Thus, the correlation between the crystallization behaviors and the tensile properties of PP/PEs blends were addressed. Furthermore, the effects of the recycling process on the chain structures, crystallization behaviors, as well as the tensile properties of recycled PP/PEs blends, are discussed.

## 2. Materials and Methods

### 2.1. Materials

The polymers chosen for this study have substantial differences in processing behavior and end-use properties. We selected the commercial virgin resins which are commonly used for packaging applications including Low-density polyethylene, LDPE (DOW LDPE 132I, Dow Chemical Company, Chicago, IL, USA), high-density polyethylene, HDPE (Marlex HXM 50100, Chevron Phillips Chemical, Woodlands, TX, USA) and polypropylene, PP (FHR Polypropylene P9G1Z-047, Flint Hills Resources, Longview, TX, USA).

The manufacturing scrap of recycled LDPE (Bapolene® 1072, Bamberger Polymers, Inc., Itasca, IL, USA), injection molding grade used for food packaging) and regrind PP (Inspire ® 6025N, Braskem, Philadelphia, PA, USA), used for blown film and thermoforming of packaging containers) were supplied by PLACON (Fitchburg, WI, USA). Incorporated together with virgin material of both grades were selected as representatives for recycled materials.

### 2.2. Sample Preparation

All materials were processed using a Leistritz (Somerville, NJ, USA) ZSE18HPe laboratory, modular, intermeshing, co-rotating twin-screw extruder and subsequently pelletized with the appropriate downstream equipment (a water-through, blown-air drier and a rotary cutter). The extrusion temperature profile was in a range of 180–220 °C from the hopper to die and the screw speed of 100 rpm. The PP/LDPE and PP/HDPE blends were prepared with the weight ratio of 100/0, 75/25, 50/50, 25/75 and 0/100, respectively.

For the mechanical properties’ measurement, the PP/LDPE and PP/HDPE blends were prepared as the tensile bars according to ASTM D638-14 using a Type 1. All specimens were injection molded using an Allrounder 320S injection molding machine of the ARBURG GmbH and Co. KG, Loßburg-Germany with a clamping force of 500 kN. This machine is equipped with a screw of 25 mm diameter and an effective screw length (L/D ratio) of 24. The mold temperature is set at 25 °C.

## 3. Characterization

### 3.1. Melt Flow Rate (MFR) Measurements

The melt flow rate (MFR) is a good technique to determine the effects of reprocessing since it is an indirect measurement of the melt viscosity of materials. It also indicates the changes in molecular weight and is widely used in the thermoplastic industry. The MFR measurements were carried out in an extrusion plastometer Series 4000 according to the ASTM D1238-10, using procedure A [24].

### 3.2. Differential Scanning Calorimetry (DSC)

The melting and crystallization behavior of PP, LDPE, HDPE and their blends were determined using a DSC (214 Polyma, NETZSCH Group, Selb, Germany) under nitrogen atmosphere. For crystallization and melting temperature measurement, PP, LDPE, HDPE and their blends were melted at 200 °C, held isothermal for 2 min, then cooled to −35 °C and heated to 200 °C again with the scanning temperature rate of 5 K/min. For isothermal analysis, all blends and pure polymers were quenched from 200 °C to an isothermal temperature between 100 to 125 °C.

For the analysis of the crystallization kinetics, the Thermokinetics software (Version 3.1, Selb, Germany) from NETZSCH Group was used (Model: n-Dimensional nucleation (Avrami–Erofeev)).

According to the rule of mixtures, the crystallinity of PP/LDPE blends was determined from the melting enthalpy $\left(\Delta H_{m}\right)$ [25]. The actual melting enthalpy of a blend is related to the enthalpy of the individual polymer and to their weight fraction *w* in the blends:

(1) $$\Delta H_{i}=\frac{\Delta H_{m\left(i\right)}}{w_{\left(i\right)}}$$

thus,

(2) $$\Delta H_{\mathrm{PP}/\mathrm{LDPE}}=\left(\frac{\Delta H_{m\left(\mathrm{PP}\right)}}{w_{\left(\mathrm{PP}\right)}}+\frac{\Delta H_{m\left(\mathrm{LDPE}\right)}}{w_{\left(\mathrm{LDPE}\right)}}\right)$$

The degree of crystallinity, χ, of PP, LDPE and their blends was calculated using the following equation;

(3) $$\chi =\frac{\Delta H_{i}}{\Delta H_{0}}\times \;100$$

where $\Delta H_{0}$ is the specific heat of melting of completely crystalline materials, $\Delta H_{0}=\;205\;J/g$, and 293 J/g for PP and PEs, respectively [6].

However, in PP/PEs blends, the resultant morphologies are not only attributed to thermodynamic factors but also to kinetic ones during subsequent crystallization. The crystallization kinetics are described in terms of the Avrami equation (Equation (4)), a common tool to describe overall isothermal crystallization (including nucleation and growth of spherulites). It can be also written as:

(4) ln(−ln(1−X(t)))=ln k(T)+n ln t

When applied for DSC analysis, it is assumed that the differential area under the crystallization curve with time corresponds to the dynamic changes in the conversion of mass from the melt phase to the solid phase. The relative crystal conversion X(t) as a function of time was calculated by the following equation:

(5) $$X\left(t\right)=\frac{X_{t}}{X_{\infty }}$$

where *X*(*t*) is the released heat until time t and $t_{\infty }$ is the total released heat by crystallization.

The Avrami index, n, is a complex exponent, which is related to the dimensionality of the growing crystals and to the time dependence of nucleation. While the crystallization rate coefficient, *k*, is the overall crystallization growth rate constant and related to the nucleation type, crystal growth and crystallization temperature. Based on these two values, the crystallization half-time, $t_{1/2}$, which is a representation of the crystallization rate, can be evaluated from:

(6) $$k=\frac{\ln2}{{\left(t_{1/2}\right)}^{n}}.$$

In this study, the crystallization kinetics of all materials were calculated based on the Avrami model using the Thermokinetics software.

### 3.3. Polarized Optical Microscopy

The phase transformation of PP, LDPE, HDPE and their blends was investigated using an Olympus IX71 polarising optical microscope (Melville, NY, USA) equipped with a 50× objective and a hot-stage. All samples were prepared as 10 μm thick films using a microtome. The films were heated between a glass slide and coverslip to 200 °C and kept for 2 min. Subsequently, the samples were quenched to an isothermal temperature between the crystallization temperatures for PP (125 °C) and LDPE (100 °C).

### 3.4. Tensile Tests

The tests were carried out according to ASTM D638-14 [26] using a Type 1 tensile bar on an Instron 5960 Dual Column Tabletop tensile test machine (Norwood, Massachusetts) with a 30 kN load cell. The crosshead was moved with a constant velocity of 500 mm/min until the specimen broke in the gauge section. The strength and the strain of the sample were determined based on the maximum force and the elongation of the tensile bar. At least five measurements were taken for each material.

### 3.5. Gel Permeation Chromatography (GPC) Analysis

The analyses were performed using a PL GPC220, equipped with DAWn Heleos-II, detector from Wyatt Technology (Santa Barbara, California). The two PLgel Olexis separating column with 300 mm × 7.5 mm, Agilent Technologies were employed. All measurements were performed with 1,2,4 trichlorobenzol stabilized with 0.1% BHT at the flow rate of 1 ml/min and the measurement temperature of 150 °C.

### 3.6. Scanning Electron Microscope (SEM) Analysis

The cryo-fracture surfaces of the PP/PEs blends were studied using the LEO 1530 scanning electron microscope (SEM, White Plains, NY, USA) at an accelerating voltage of 5 kV. All fractured samples were etched with sulfuric acid at 80 °C for 2 h. Prior to investigation, all samples were sputter-coated with gold.

## 4. Results and Discussion

### 4.1. Melting and Crystallization Temperatures

According to the data available in the literature as well as the one presented in this study, PP, LDPE and HDPE crystallize separately, and both polymers can have a mutual effect on the process of crystallization, and the morphology of the blends [12].

**Figure 1:**
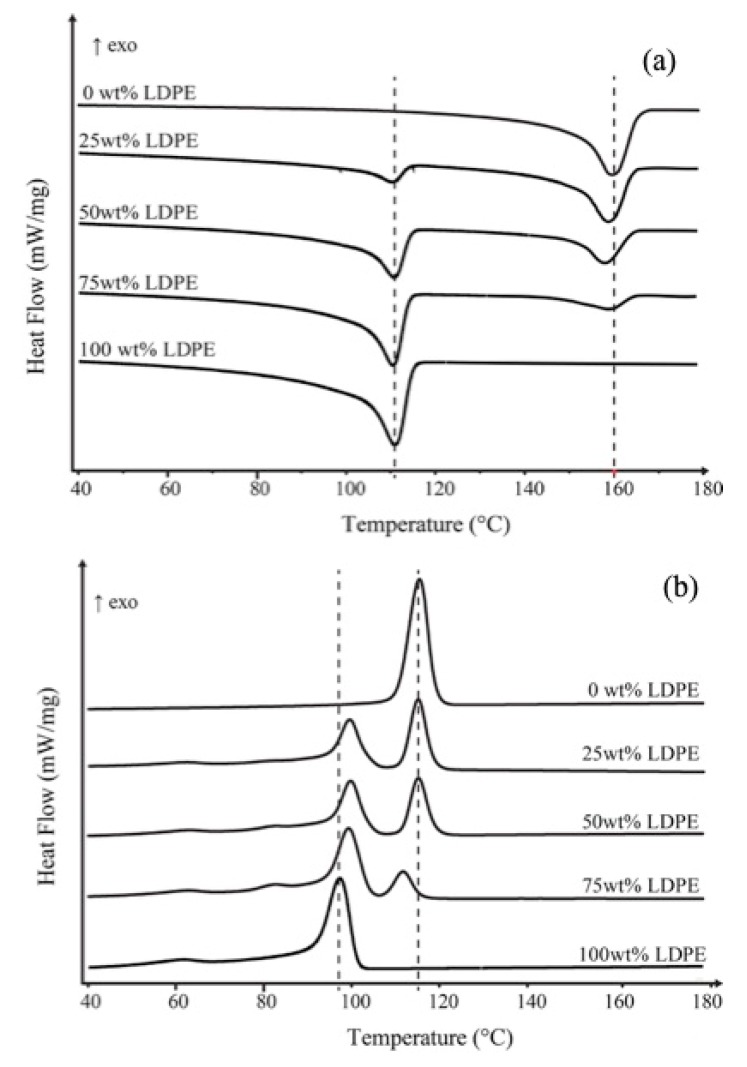
(**a**) Melting temperature (*T*_m_) from the 2nd heating scan and (**b**) crystallization temperature (*T*_c_) of polypropylene (PP), low-density polyethylene (LDPE) and their blends at different compositions.

Figure 1a shows the DSC heating curves of PP, LDPE and their blends at different compositions. The two endothermic peaks in the temperature range corresponding to the melting temperature of the individual polymers. The melting temperature of PP and LDPE does not significantly depend on the composition of the blends. As shown in Figure 1b, the crystallization temperature of LDPE in PP/LDPE blends remarkably shifts to higher temperature relative to the crystallization peak of pure LDPE and remains almost constant in all blend compositions. However, the crystallization temperature of PP in PP/LDPE blend with 75 wt % of LDPE content shifts to lower temperatures, which indicates a reduction in the perfection of the formed crystallites.

**Figure 2:**
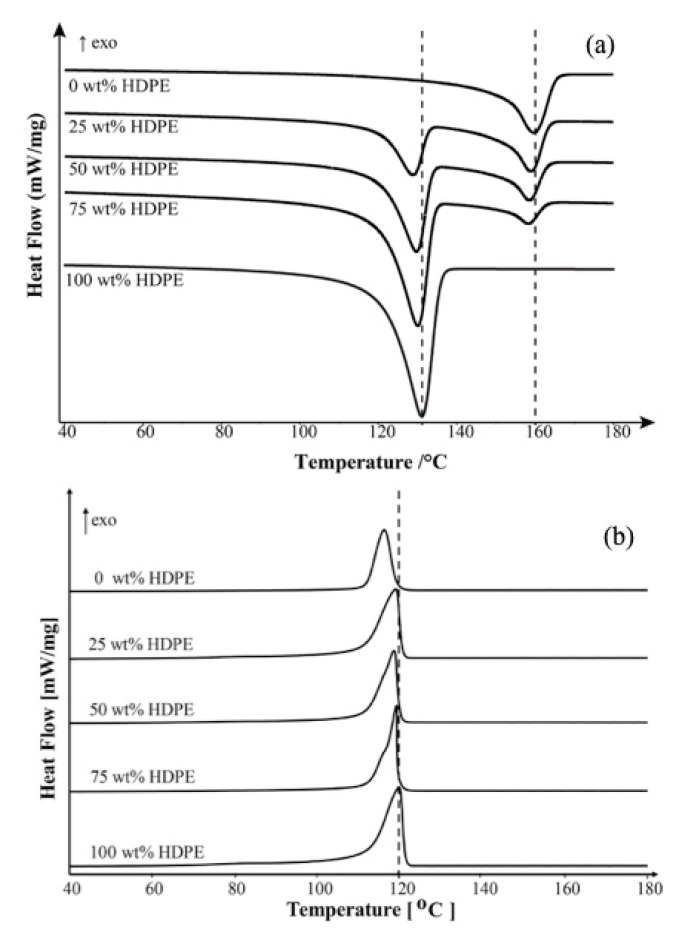
(**a**) Melting temperature (*T*_m_) from the 2nd heating scan and (**b**) crystallization temperature (*T*_c_) of PP, high-density polyethylene (HDPE) and their blends at different compositions.

For PP/HDPE blend, the *T*_m_ of HDPE in all blend compositions remains almost constant temperatures relative to that of pure HDPE (~131 °C) as shown in Figure 2a. However, the crystallization behavior of PP/HDPE blends is complex compared with the melting behavior. As shown in Figure 2b, the crystallization peak of blends shifts to the higher temperature, which is close to the crystallization temperature (*Τ*_c_) of HDPE. Even though PP/HDPE blend systems seem to show only one *Τ*_c_, however, the bimodal behavior is more pronounced at higher HDPE loading blend (75 wt % HDPE). The bimodal crystallization behavior could be associated with partially miscible behavior. Also, a single crystallization peak of the blend with small HDPE loading could be postulated that the PP was miscible with HDPE at elevated fraction and temperature. A single crystallization peak may be due to a very close crystallization temperature of the individual polymers; 116.2 °C for PP and 117.6 °C for HDPE. Also, the one crystallization peak possibly implies co-crystallization of the materials, which leads to miscibility and compatibility of the blends.

### 4.2. Crystallinity

**Figure 3:**
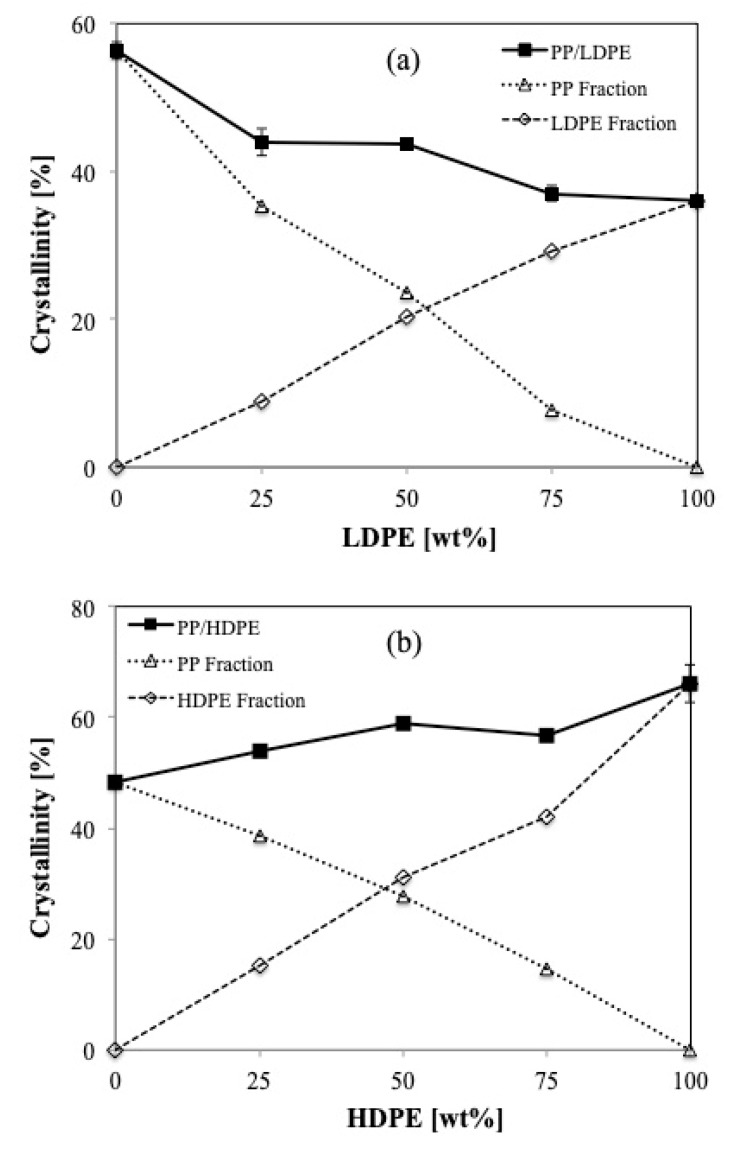
% Crystallinity of (**a**) PP/LDPE blends and (**b**) PP/HDPE blends at different blend compositions.

Figure 3a presents the crystallinity of PP/LDPE blends both total and separately in the blends. It can be seen that the crystallinity of PP and LDPE in the blends varies depending on their composition. In the case of high viscosity polymers, the increase of LDPE (lower viscosity) content decreases the degree of crystallinity of the blend. Concerning an individual component, it is seen that the crystallinities of both LDPE and PP in the blends exhibit an almost linear relationship with its concentration.

Similarly, Figure 3b presents the total crystallinity of PP/HDPE blends both total and separately in the blends. The more linear structure of HDPE results in more crystallinity compared to those of PP. The larger the HDPE contents, the higher crystallinity of the blends. Remarkably, a drop of the total crystallinity of PP/HDPE blends appears when the blend contains 75 wt % HDPΕ, which could be related to the deviation in crystallization behavior as well as the crystal structure of the blend.

### 4.3. Crystal Structures

The crystal structures and morphology of PP, LDPE, HDPE and their blends were captured using the polarized optical microscope. From the DSC results, it is known that PP starts crystallizing at 125 °C while LDPE and HDPE begin at 100 and 124 °C, respectively.

**Figure 4:**
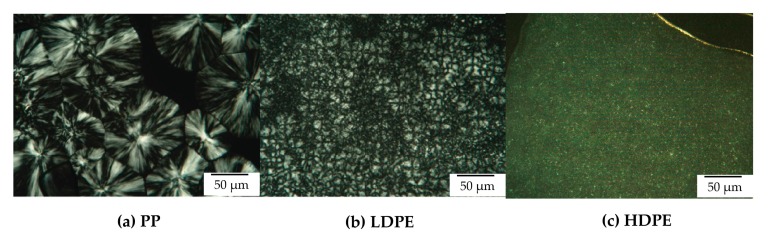
Polarizing optical microscopy of (**a**) LDPE after isothermal crystallization at 100 °C for 20 min, and (**b**) PP after isothermal crystallization at 125 °C for 20 min and (**c**) HDPE after isothermal crystallization at 124 °C.

PP is a semi-crystalline thermoplastic polymer with a high degree of crystallinity due to its regular chain structure. In general, PP can crystallize in a wide range of spherulite dimensions (from 10–50 μm to 280–370 μm) depending on the crystallization conditions as well as the presence of nucleating agent [27,28]. LDPE and HDPE crystals, in general, consist of ethylene units in a chain fold structure. Thus, the segment length of an ethylene unit can limit the lamella thickness. From this study, it is visible that LDPE crystals are much smaller in size compared to PP crystals. The crystal sizes are about 25 and 100 μm in diameter for LDPE crystals and PP crystals, respectively. However, the crystal sizes of HDPE are much smaller than those of PP and LDPE as can be observed in Figure 4.

**Figure 5:**
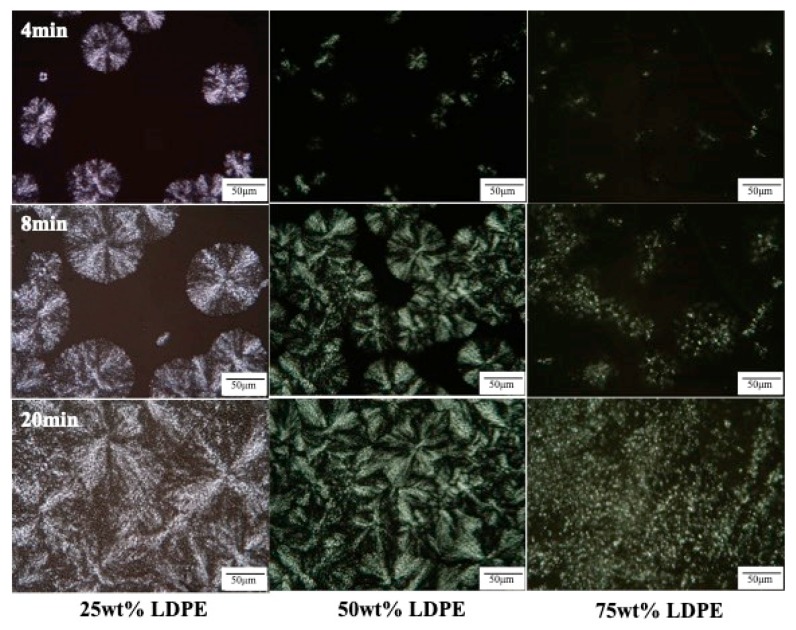
Polarizing optical microscopy of the nucleation and growth process of PP/LDPE blends containing 25 wt % LDPE, 50 wt % LDPE and 75 wt % LDPE during isothermal crystallization at 125 °C.

In this study, each blend was crystallized isothermally at 125 °C, where PP could crystallize. Figure 5 shows the nucleation and growth process of PP/LDPE blends during the isothermal crystallization at 125 °C from 4 to 20 min. The spherulites of PP in blends were not as sharp as in the virgin PP, but they could still be readily distinguished. During crystallization, the PP in the blend crystallizes at almost the same rate as pure PP. A larger LDPE content resulted in a reduction of the average spherulite size of PP. Possibly, PP should be somewhat soluble in the melted LDPE, which seems to be consuming the remaining PP dissolved in the matrix, preventing bridging growth [29]. This can also be concluded from the shifting of *T*_c_ of PP to a lower temperature with more substantial LDPE contents.

The crystallization rate of PP was found to be slightly slower at larger LDPE contents. As can be seen from Figure 5 at 4 min, the PP spherulite in the 75 wt % LDPE blend developed slower than that of PP spherulites in the 50 wt % LDPE and 75 wt % LDPE blends, respectively. Even though the PP crystals in the blend of 25 wt % LDPE nucleated faster than that of the blend with 50 wt % LDPE, it should be noted that once the PP crystals in the blend of 50 wt % LDPE were nucleated, they grew faster. This can be seen at 8 min. Some crystals nucleated at a much lower rate and then grew faster afterward.

**Figure 6:**
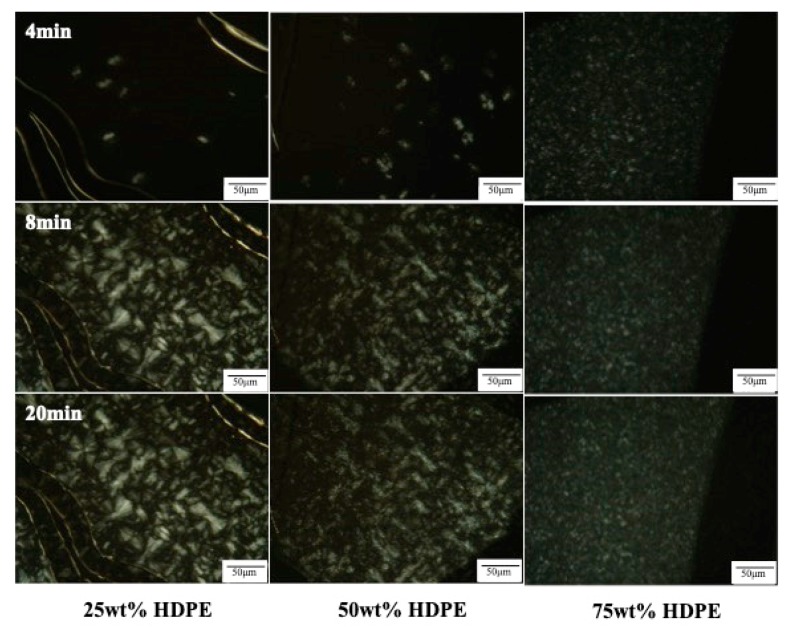
Polarizing optical microscopy of the nucleation and growth process of PP/HDPE blends containing 25 wt % HDPE, 50 wt % HDPE and 75 wt % HDPE during isothermal crystallization at 125 °C.

In contrast, the opposite effects are found for PP/HDPE blends. The larger the HDPE contents, the faster the crystallization rate as can be seen in Figure 6. Since the crystallization temperatures of PP and HDPE overlap, both PP and HDPE could crystallize, increasing the crystallization rate. The overall crystallization rates are accelerated as the higher HDPE contents are loading.

### 4.4. Crystallization Kinetics

**Figure 7:**
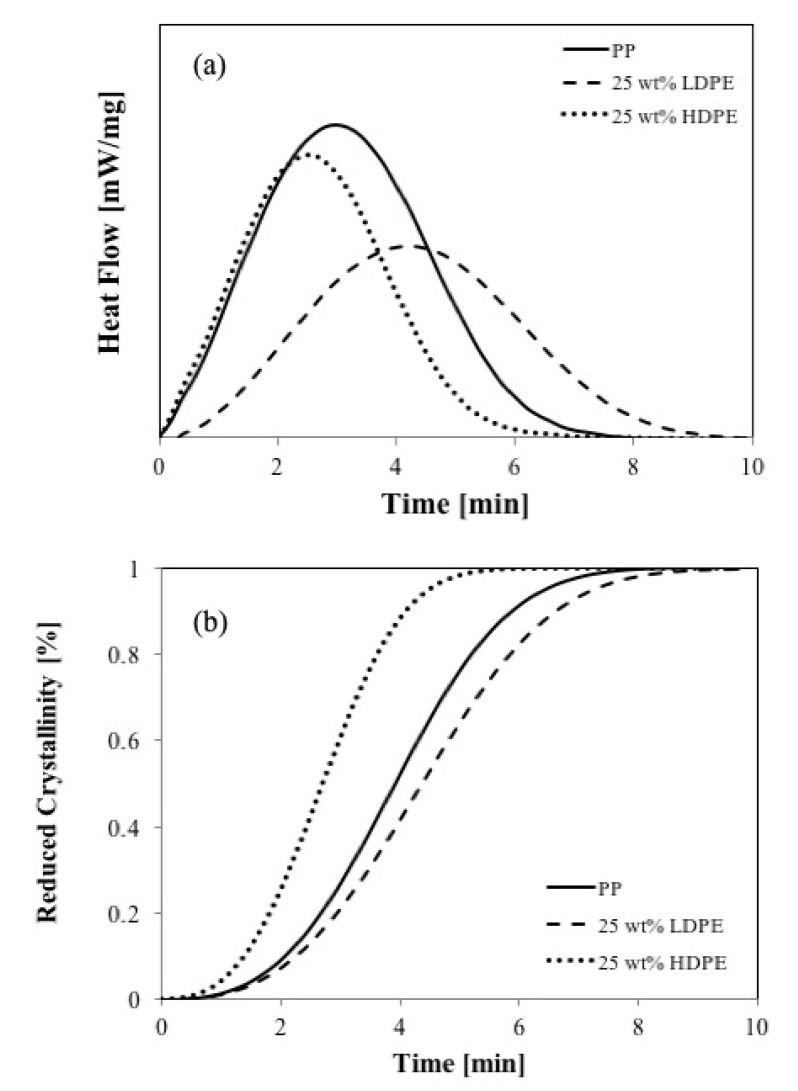
(**a**) Differential scanning calorimetry (DSC) crystallization curves and (**b**) reduced crystallinity obtained at 125 °C for PP (solid line), PP/LDPE containing 25 wt % of LDPE (dash line) and PP/HDPE blends containing 25 wt % of HDPE (dot line).

Figure 7a shows the crystallization behaviors, and Figure 7b shows the comparison of crystalline fraction development in PP/LDPE and PP/HDPE at the same concentration of LDPE and HDPE (25 wt %), respectively. The solid line represents the crystalline fraction of pure PP at the same isothermal crystallization process.

Based on the Avrami equation, the values of the Avrami exponent, *n*, varied slightly but they were all close to 2, indicating that the crystallization process was heterogeneous and took place within two dimensions. The growth of spherulites was confined in two dimensions although initial nucleation and crystallization may take place in three dimensions because the specimens were 10 μm in thickness and were sandwiched between glass slides and coverslips. The most dramatic increase in the Avrami index, *n*, can be obtained by increasing the LDPE content since the morphology can also be significantly altered by confinement effects or interference from the LDPE melt. The growing PP spherulites may occlude the droplets of LDPE. Theoretically, the difference in interfacial energy decides whether the droplets of LDPE were engulfed or rejected by the growing PP spherulites [28]. The crystallization kinetic parameters of all blends are shown in Table 1.

From the crystallization behavior studies, which include the kinetics of crystallization as well as the crystal morphology from the optical microscope, we found that the overall crystallization rate of PP was sharply reduced by the addition of LDPE, the more branched structure. However, the opposite results were found for the case of linear chain HDPE. Consequently, the branched-chain structure seems to affect the nuclei density. However, from this study, it can be said that both nucleation and diffusion processes controlled the overall crystallization rate.

The MFR seems to affect the crystallization haft-time, as shown in Table 1. The addition of LDPE and HDPE into PP showed different effects on the MFR of the blends. The MFR of PP/HDPE blends decreased with increasing the HDPE content, and all blend compositions have the MFR within the limits of the pure components; PP and HDPE. Surprisingly, the MFR of PP/LDPE increased with increasing LDPE loading, which is even higher than the MFR of the pure components; LDPE and PP. It is possible that LDPE acts as a plasticizer for the PP molecules and enhances the mobility and diffusion of PP in the blends.

### 4.5. Effect of Recycling Process

Due to the large differences in structure and behavior, for recycled materials, the results presented in this study will be limited to PP, LDPE and their blends. Some results of PP/HDPE blends will be shown to confirm the applicability of methods or results where appropriate. Thus, the manufacturing scraps of recycled LDPE (Bapolene® 1072) and regrind PP (Inspire ® 6025N), which will be called as LDPE1 and PP1 were selected as representatives.

**Figure 8:**
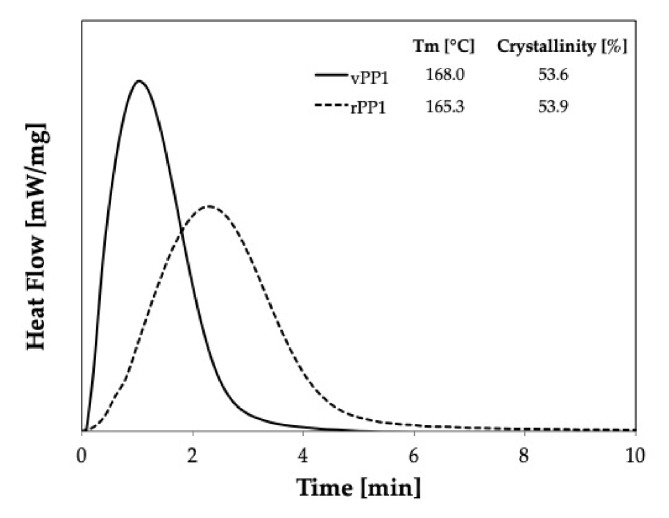
Crystallization curves obtained at 139 °C for virgin material (vPP1), and recycled material (rPP1).

Figure 8 shows the comparison of isothermal crystallization behavior of virgin material (vPP1, solid line) and recycled material (rPP1, dash line) at 139 °C. It is seen that the crystallization behavior of recycled PP deviates from that of the virgin material. The broader shape indicates that rPP1 needs more time to crystallize and that the crystal geometry could be different.

**Figure 9:**
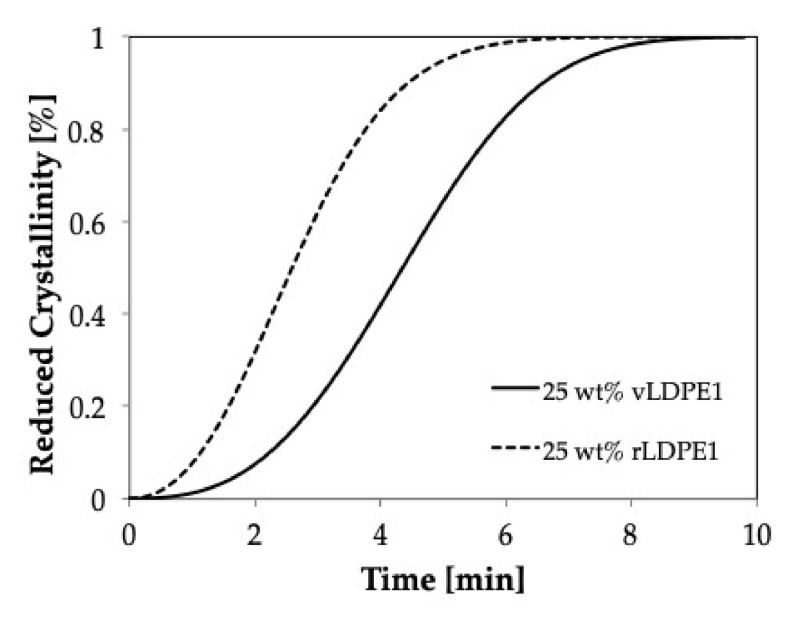
Crystallinity obtained at 139 °C for virgin material (vPP1/vLDPE1) and recycled material (rPP1/rLDPE1) blends containing 25 wt % of vLDPE1 and rLDPE1, respectively.

Figure 9 shows the isothermal crystallization curves for vPP1, and vPP1/vLDPE1 blends at 139 °C, a temperature at which the only vPP1 can crystallize. These results imply a delay in the crystallization and the imperfection of the crystal formation in the recycled blends.

The effect of recycling process on the crystallization behavior of PP/LDPE blends is shown in Figure 9. The recycled blend (dash line) shows a higher rate of crystallization compared to that of the virgin blend (solid line), which may be caused by the shorter polymeric chain resulting from the degradation during the recycling process [14]. Furthermore, the recycling process affects the shape of the crystals (rPP1), which become more spherical. The smaller the value of *k* and the higher the half-time value, the slower is the rate of crystallization.

It can be seen that the recycling process resulted in the broader of the crystallization peak. Moreover, the nucleation of rPP1 is much slower than that of vPP1, as reported in Table 2. Furthermore, a significant decrease in *M*_w_ of virgin PP1 (vPP1) from 481,000 to 354,150 g/mol for recycled PP1 (rPP1) was observed. Recycled PP1 (rPP1) is the regrind material received out of the production stream, resulting from the dilution of 70% recyclate with 30% virgin material. Thus, the properties can be influenced by highly degraded fractions, which have shorter polymer chains than the virgin material. The shorter chains have a lower molecular weight and thus, affect the mechanical performance of the material. The observed lowering in the molecular weight (*M*_w_) indicates that chain scission occurred during the recycling process. The lower polydispersity index shows that the chain scission was mostly affecting the longest chains, while the lower limit was not affected. Thus, the molecular chain of rPP1 is shorter, and the molecular weight distribution is narrower, leading to the smaller k value and lowering the crystallization rate as shown in the increasing of the crystallization half-time.

From the crystallization behavior studies, which include the kinetics of crystallization as well as the crystal morphology from the optical microscope, it is clearly shown that the overall crystallization rate of PP was strongly reduced by the addition of LDPE for both the high-viscosity system (PP/LDPE and PP/HDPE) and low-viscosity system (vPP1/vLDPE1). Furthermore, consistent behavior was observed in the blend system of recycled material (rPP1/rLDPE1). It can be concluded that the reduction in the overall rate is attributed to a decrease in the nucleation density. The decrease in nucleation density was found predominantly caused by the nuclei migration from PP to PE, which was in turn caused by an interfacial energy difference [19].

In general, polymer degradation during processing operations can cause changes in molecular weight and molecular weight distribution in polymers. The MFI indicates changes in molecular weight but is rather insensitive to changes in molecular parameters such as molecular weight distribution. In the case of viscosity, the sensitivity to molecular weight distribution changes is seen in the very low and very high shear rate ranges. MFI has been used to estimate the extent of thermal and shear degradation of polymeric material as it can be readily measured on a simple and inexpensive melt flow indexer. The recycled blend shows a higher MFI at the blend composition of less than 50 wt % of LDPE while the lower MFI can be observed at the high LDPE contents (>50 wt %). Therefore, it can be concluded that at small LDPE contents, the MFI of the matrix PP dominates the change in MFI of the blend. Vise Versa, at higher LDPE content, when the LDPE presents the matrix, the MFI of the blends are indeed governed by LDPE. The low-viscosity materials (high MFI) show a faster crystallization rate as well as shorter crystallization half-time compared to the high-viscosity materials.

### 4.6. Mechanical Properties

**Figure 10:**
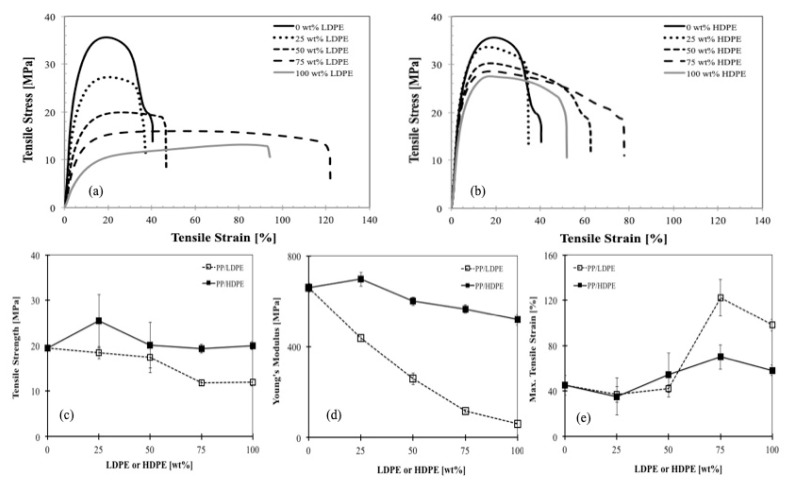
Tensile properties: (**a**) stress-strain curves of PP, LDPE and their blends at different compositions (u_0_ = 500 mm/min), (**b**) stress-strain curves of PP, HDPE and their blends at different compositions (u_0_ = 500 mm/min), (**c**) tensile stress, (**d**) Young’s modulus and (**e**) tensile strain at maximum stress for PP/LDPE (dash line) and PP/HDPE (solid line) as a function of blend composition.

Figure 10a presents the stress-strain curves for PP, LDPE and their blends at different compositions. The tensile stress-strain curves are dependent on their composition. As expected, the elongation of PP was improved by adding the more ductile LDPE. The larger the content of LDPE, the higher is the strain at break. The same behaviors were observed for the PP/HDPE blends as shown in Figure 10b. The tensile strength, Young’s modulus and maximum tensile stress for such PP/LDPE and PP/HDPE blends are depicted in Figure 10c–e, respectively. It can be seen that Young’s modulus and the tensile strength of PP/LDPE and PP/HDPE blends followed the same trends. However, a distinct improvement in Young’s modulus and tensile strength were found in PP/HDPE blend with 25 wt % of HDPE. One reason could be attributed to the co-crystallization of PP and HDPE (exhibit one crystallization peak), and the higher degree of crystallinity, which again results in an increased modulus.

**Figure 11:**
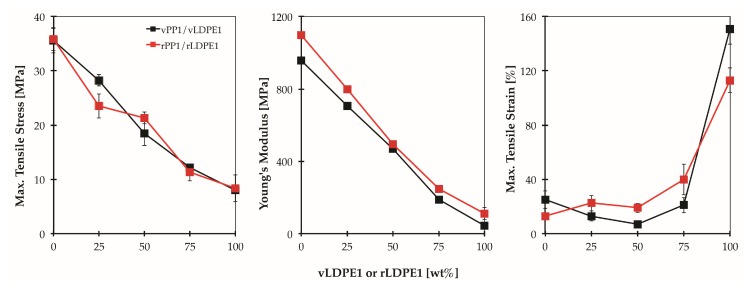
Tensile stress [MPa], Young’s modulus [MPa] and tensile strain [%] for vPP1/vLDPE1 and rPP1/rLDPE1 as a function of blend composition.

Figure 11 shows the tensile properties of the blends prepared from virgin materials (vPP1/vLDPE1), and recycled materials (rPP1/rLDPE1), respectively. The tensile properties of rPP1 show only a slight change compared to those of virgin material (vPP1/vLDPE1). These results indicate the potential for recycling the PP/LDPE blend with consistent mechanical performances.

### 4.7. Blend Morphology

**Figure 12:**
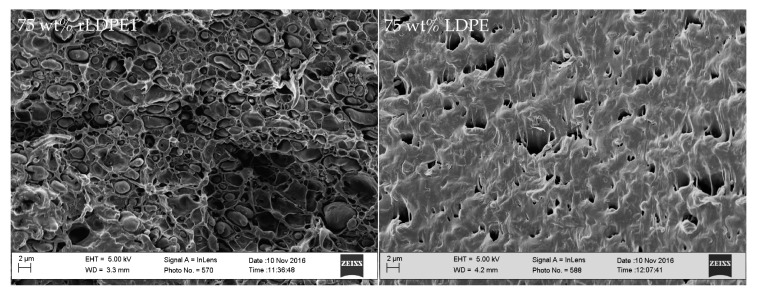
SEM images for vPP1/vLDPE1 (left) and PP/LDPE (right) at 25 wt % of LDPE composition, sulfuric etching at 80 °C for 2 h.

Figure 12 presents the SEM micrographs for the low-viscosity blend (rPP1/rLDPE1, MFI ratio = 10) in comparison with the high-viscosity blend (PP/LDPE, MFI ratio = 1) of 75 wt % LDPE. It is seen that the polymer pairs with different viscosities and MFI ratios, in which related to the different chain structures and degree of entanglement, developed different phase morphologies. The rPP1/rLDPE1 blend shows a droplet structure, small holes as well as droplets of material being dispersed in the matrix. However, sharp borders at the dispersed phase sites and no evidence of PP-LDPE interpenetration are observed. Since rLDPE1 has a much lower viscosity than rPP1 in this blend, holes and droplets may be attributed to LDPE particles, which are entirely surrounded by PP. The lack of interfacial adhesion between PP and LDPE phases leads to the decreasing in mechanical strength of the blend as presented in Figure 10 and Figure 11.

Polymer pairs and blend composition affect the results of the short-term tensile test. The higher maximum tensile stress of PP can be explained by the short rigid methyl group, which is attached to every second carbon atom of the polymer backbone. The methyl group restricts rotation of the chain, which results in a higher degree of crystallinity while producing a stronger and less flexible material. Thus, already a small content of this brittle material decreases the ability of elongation significantly, which is shown by the considerable drop of maximum tensile strain between 0 and 25 wt % LDPE. Such behavior can be also observed all blends.

The PP/LDPE in this study shows extremely improved tensile properties at 75 wt % LDPE content, which imply partial miscibility or partial compatibility of the blend. Similar behavior is also observed in PP/HDPE blend. Incorporating with the crystal structure results, it could be said that the PP/PEs blends showed a dynamic incompatible behavior that has been attributed to the different crystalline structures of the two components, PP and PEs. Even though it took longer time to crystallize, blends with 25 wt % of LDPE and HDPE crystallized with the large size of crystals. This could be causing a heterogeneous structure with weak interconnections, leading to modest maximum tensile strain values. As the LDPE and HDPE content increase, the blends getting crystallized into aggregates of smaller sizes in which well interconnections between the crystals, leading to the tensile strain enhancement. The PP/PEs blends with a high level of compatibility are expected to offer advantages for a wide range of applications, including the applications of the individual polymers.

## 5. Conclusions

The crystallization behavior studies, which include the kinetics of crystallization as well as the crystal morphology from the optical microscope, clearly showed that the addition of long branches LDPE strongly reduced the overall crystallization rate of PP. The reduction in the overall rate is mainly attributed to a decrease in the nucleation density. The decrease in nucleation density was found predominantly caused by the nuclei migration from PP to LDPE. Accordingly, the imperfection of PP crystals in the blends resulted from LDPE droplets engulfed in the PP spherulites, which can also be observed from the shifting of *T*_c_ of PP to a lower temperature with larger LDPE contents. The crystallization rate of PP was found to be slightly slower at larger LDPE contents. Even though the PP crystals in the blend with 25 wt % LDPE nucleated faster than that of the blend with 50 wt % LDPE, it should be noted that once the PP crystals in the blend of 50 wt % LDPE were nucleated, they grew faster. This can be seen in the optical microscopic images for the isothermal crystallization at 8 min as well as the overall crystallization rate, which is represented in the form of reduced crystallinity versus time. Some crystals nucleated at a much lower rate and then grew faster afterward. However, in the case of PP/HDPE blend in which the crystallization temperatures overlap, resulting in an increase of the crystallization rate. Moreover, the more LDPE and HDPE contents, the smaller crystal sizes propagated in the blends. The Avrami coefficient, *n*, depends on the shape of the crystal: spherical (*n* = 3); disk-shaped (*n* = 2); rod-shaped (*n* = 1). This study shows that the shape of the crystal and the crystallization rates seemed to affect the tensile properties of the blends. These results are observed for the blends of PP and HDPE (short branched structure). The PP/HDPE blend with 75 wt % HDPE, which *n* = 1.69 and had a prolonged crystallization rate, showed a significantly improved on the elongation property. Accordingly, the slower crystallization rate resulted in the tensile properties improvement.

The mechanical properties are in good agreement with the crystallization study and show that PP/LDPE with 75 wt % LDPE and PP/HDPE with 75 wt % HDPE are partially compatible. Particularly the elongation at maximum strain is considerably improved while the tensile stress at break and Young’s modulus are not lower than expected. Thus, the recycled PP/PEs blends can be expected to be able to serve in a wide range of application. This is depending on the degree of miscibility or compatibility, which is related to the desired properties of the products as well as the processability of the materials. According to this study, the properties of recycled PP/PE blend (rPP1/rLDPE1) remains unchanged until the composition of rLDPE1 reach 25 wt %, which means that rPP1/rLDPE1 can be used similarly as the pure recycled PP. For example, the recycled blends could be used again in injection molded or blow-molded applications to make bottles or containers. Furthermore, the outdoor decking, fencing and picnic tables can be manufactured from the recycled PP/PEs blends as well as pipe, films or sack bags.

## Figures and Tables

**Table 1:** Avrami index (*n*), overall crystallization rate constant (*k*), and crystallization half-time obtained during isothermal crystallization of PP, LDPE, HDPE and their blend at isothermal crystallization temperatures (125 °C) at the cooling rate of 25 K/min.

Materials	MFR [g/10 min]	Half-Time, *t*_1/2_ [min]	*n* [-]	*k* [min^−1^]
PP (0 wt % LDPE)	0.66	3.24	2.42	0.2655
PP/LDPE (25 wt % LDPE)	0.71	4.58	2.73	0.1908
PP/LDPE (50 wt % LDPE)	0.89	4.68	2.65	0.1862
PP/LDPE (75 wt % LDPE)	0.91	N/A	N/A	N/A
LDPE (100 wt % LDPE)	0.72	N/A	N/A	N/A
PP/HDPE (25 wt % HDPE)	0.45	2.73	2.36	0.3143
PP/HDPE (50 wt % HDPE)	0.32	2.27	2.54	0.3808
PP/HDPE (75 wt % HDPE)	0.23	N/A	N/A	N/A
HDPE (100 wt % HDPE)	0.15	N/A	N/A	N/A

**Table 2:** Weight average molecular weight (*M*_w_), molecular weight distribution (*M*_w_/*M*_n__)_ and kinetic parameters for the isothermal crystallization of vPP1 and rPP1 at 139 °C at the cooling rate of 25 K/min.

Materials	MFR [g/10min]	*M*_w_ [g/mol]	*M*_w_/*M*_n_	Crystallization Temperature [°C]	Half-Time, *t*_1/2_ [min]	n [-]	*k* [min^−1^]
vPP1	2.76	414,933	3.15	139	1.52	1.93	0.5436
rPP1	6.10	333,467	2.46	139	2.21	2.24	0.3844
